# Towards understanding the effects of heat and humidity on ageing of a NASA standard pyrotechnic igniter

**DOI:** 10.1038/s41598-019-46608-8

**Published:** 2019-07-15

**Authors:** Juyoung Oh, Seung-gyo Jang, Jack J. Yoh

**Affiliations:** 1 Department of Mechanical and Aerospace Engineering, Seoul National University, Seoul, 08826 South Korea; 2 Agency for Defense Development, Daejeon, 34060 South Korea

**Keywords:** Chemical engineering, Actuators

## Abstract

Ageing of pyrotechnic substance, primarily fuel oxidisation, can cause changes in composition that degrade their performance. This study investigates the effect of ageing on zirconium potassium perchlorate (ZPP), a widely used NASA Standard Initiator. Although prior studies have investigated the effects of accelerated ageing on ZPP, this is the first to conduct kinetic analyses at different relative humidity (RH) levels. Here, both thermal and kinetic analyses are conducted for a variety of hygrothermal ageing cases in order to replicate the natural ageing process. X-ray photoelectron spectroscopy (XPS) results reveal that oxidant levels drop and zirconium dioxide levels rise as ZPP ages. Lower heats of reaction and increases in activation energy were also observed under the RH conditions. Calculations using van’t Hoff equation indicate that moisture shortened the lifespan of the unaged ZPP up to about 85% under extreme RH conditions, while significantly deteriorating the heat of reaction, sensitivity, and thus increased the risk of a misfire.

## Introduction

As energetic materials age, their intended performance is known to significantly degrades due to oxidisation, hydrolysis, chemical reactions or structural deformations in their chemical constituents. Ageing is usually a slow process, and its effects often take several decades to become apparent in military applications. Ageing studies have therefore concentrated on examining samples that have been stored for extended periods of more than twenty years^1–3^. That said, some researchers have attempted to predict the effects of ageing using the accelerated ageing techniques^4–13^, which can estimate the remaining life time of aged samples^9,14^.

So far, however, these studies have not considered the fact that, in real environments, humidity is an essential component within the storing ambient. Even if the pyrotechnic substances are air-tight sealed, their exposure to an external environment is simply inevitable prior to such sealing process^15^. In addition, the seals on energetic materials can sometimes be damaged during the long-term storage. Therefore, it is a concerning factor whether relative humidity (RH) plays an essential role in the ageing of pyrotechnic substance.

**Figure 1:**
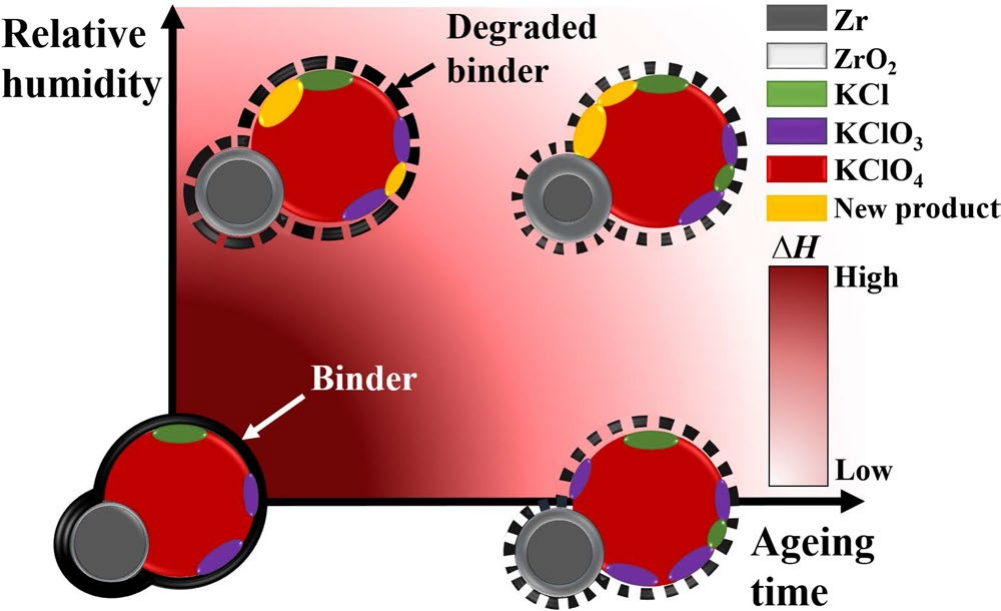
Illustration of the relationship between relative humidity and ageing time for ZPP. The binder’s performance often degrades over time and with increasing relative humidity. As the binder degrades, it increasingly exposes the surface area of ZPP to the ambience. High relative humidity levels cause the Zr to oxidise, forming a ZrO_2_ coat. Ultimately, ageing causes the oxidants to decompose and the fuel to onse, both of which result in reduced ZPP performance.

In order to understand the effects of ageing on pyrotechnic performance, the present study examines zirconium potassium perchlorate (ZPP), a NASA Standard Initiator that is thermally stable and known to have a long shelf-life^15–17^. In ZPP, zirconium (Zr) is the fuel and potassium perchlorate (KClO_4_) acts as the oxidising agent, while structurally bound with a Viton b^18^. Energetic materials are designed to achieve their performance targets, such as blasting pressure or light, sound or heat emission, by generating redox reactions between the fuel and oxidants^19^. However, as ZPP ages, its composition changes which results in the changes of thermodynamic characteristics and possible performance degradation, as shown in Fig. 1.

To replicate the effects of ageing on pyrotechnic substances, work has been done to establish the effects of thermal and humidity conditions^20–29^. Most recently, Lee *et al*.^22^ investigated ZPP’s ageing mechanism under a 100% RH condition, where they focused primarily on the oxidation of Zr during such a limited aging condition without providing the relevant kinetic analyses of aging ZPP associated with particular reaction processes.

The current study investigates the chemical changes of the oxidiser, reaction kinetics, detailed thermal reaction processes, and lifespan, which have not been discussed in prior studies. In particular, the study aims to provide in-depth details into the effect of ageing on ZPP in terms of both thermal and hygrothermal ageing. Three humidity conditions (30%, 70%, 100% RH) and one thermal condition (0% RH) are considered. The results of both kinetic and thermal analyses under these four ageing conditions are discussed by utilising differential scanning calorimetry (DSC). It also examines the changes in both the oxidants and fuel due to ageing by utilising X-ray photoelectron spectroscopy (XPS) for surface analyses^30^. Finally, the van’t Hoff equation is used to confirm the relationship between the heat of reaction and lifespan of ZPP. This ultimately results in a characterisation of the changes in the property of ZPP due to varying ageing conditions. The high-RH conditions gave rise to a significant reduction in the lifespan of ZPP.

## Results

### Qualitative XPS analyses

XPS was used to observe the changes in ZPP composition due to thermal and hygrothermal ageing. Since these experiments focused purely on confirming ageing-related phenomena, particular ZPP specimens were designated as representatives of the different ageing groups; these are indicated by ‘O’ symbols in the ‘Utilised for XPS’ column in Table 1. These qualitative XPS analyses showed that the unaged ZPP sample (Sample 0) exhibited six main peaks (Zr, Cl, K, O, F and C), coinciding with the expected ZPP composition (see Supplementary Fig. S1).

**Table 1:** Conditions under which the ZPP samples were aged.

Sample label	Ageing conditions			Utilised for XPS	Ageing type
	Temperature	Relative humidity	Ageing duration		
0	—	—	—	O	Unaged
1	71 °C	0%	4 months	O	Thermal only
2			6 months	—	
3			8 months	O	
4			12 months	O	
5		30%	2 weeks	O	Hygrothermal
6			4 weeks	—	
7			8 weeks	O	
8			16 weeks	—	
9		70%	2 weeks	O	
10			4 weeks	—	
11			8 weeks	O	
12			16 weeks	—	
13		100%	2 weeks	O	
14			4 weeks	O	
15			6 weeks	—	
16			12 weeks	O	

### Quantitative XPS analyses

**Figure 2:**
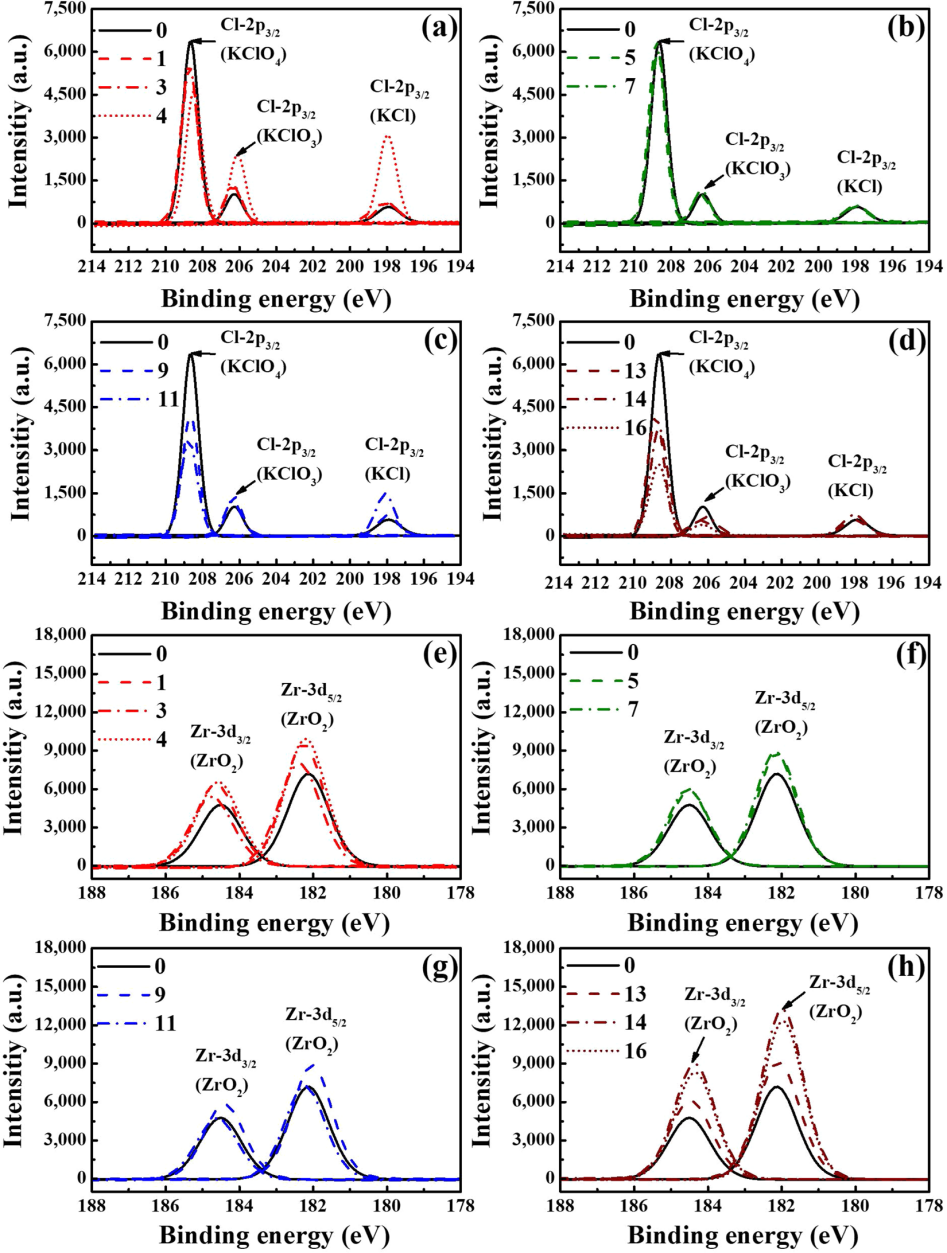
XPS spectra of the oxidants (**a**–**d**) and fuel (**e**–**h**) under different ZPP ageing conditions: (**a**,**e**) thermal ageing, (**b**,**f**) 30% RH, (**c**,**g**) 70% RH and (**d**,**h**) 100% RH. These show that, as the ageing duration and RH increased, the ZPP’s KClO_4_ content tended to decrease and the amount of ZrO_2_ increased.

Figure 2 shows how both the RH level and ageing duration affected the ZPP samples. Small amounts of KClO_3_ and KCl were also observed in the XPS results, even though the unaged ZPP sample consisted of more than 99% pure KClO_4_. Thus, these results also show the relationships between KClO_3_ (206.50 eV) and both KCl (198.00 eV) and KClO_4_ (208.70 eV).

As Fig. 2(a–d) show, the composition of the KClO_4_ oxidiser varied depending on the ageing conditions. In the thermal ageing case, a substantial number of KClO_4_ molecules decomposed into potassium chlorate (KClO_3_) and potassium chloride (KCl) when the ZPP had aged by more than a year (Fig. 2(a)), indicating that thermal ageing can trigger ZPP’s oxidiser to decompose into KClO_3_ and KCl. In the hygrothermal ageing case, on the other hand, at low humidity levels (30% RH) have no significant effect on the ZPP composition (Fig. 2(b)). By contrast, at higher RH levels (70% and 100%), KClO_4_ decomposed rapidly as the ageing period increased (Fig. 2(c,d)). At 100% RH, in particular, KClO_4_ decomposed without creating KClO_3_ or KCl, implying that ageing at high-RH levels may lead to the production of new products.

We have mainly focused on the five atoms, ‘Zr’, ‘Cl’, ‘O’, ‘K’, and ‘C’ to observe any changes in the molecular composition of the metal fuel and oxidants of the aged ZPP samples. All compounds related to ‘Cl’ which can be found on the surface of ZPP were represented in Fig. 2(a–d). In the compounds associated with ‘K’, only the reduced concentrations in KClO_4_ were found without any increasing trend of the decomposition products. No components were found in the XPS results to represent the new products. XPS technique can detect the surface composition for all signals except ‘H’ atom. For this reason, Cl-based molecules combined with hydrogen were not found. Thus, it can be assumed that the oxidants were produced as the ‘new products’ combined with ‘H’ atoms due to hydrolysis reaction during the hygrothermal ageing process.

Figure 2(e–h) show the presence of oxidised fuel. Since Zr is reactive and sensitive in its pure form, it is naturally coated with a thin oxide layer at the moment of exposure to the atmosphere^31^. These XPS results demonstrate the growth of zirconium dioxide (ZrO_2_), indicating that, as it ages, ZPP is reacting with either oxygen in the air or oxidants to form ZrO_2_. The ZrO_2_ content (182.20 and 184.50 eV) increased rapidly with both the RH level and thermal duration. In particular, the 100% RH samples showed the highest ZrO_2_ content among the four ageing groups, which is consistent with the previous study^20,25^, demonstrating that the limiting RH condition has a significant effect on the material’s explosive characteristics.

In summary, the XPS analyses indicate that ageing had a substantial effect on the samples aged for prolonged durations and at high-RH levels, which was already illustrated in Fig. 1. These changes became more pronounced in both fuel and oxidants as the RH level increased from the quantitative data based on XPS results (see Supplementary Fig. S2). When compared to sample #0, Fig. S2 shows the growth of oxide layer on zirconium for each sample and illustrates how much of oxidants are decomposed by the ageing. Thus, these results imply that the RH level and ageing duration have a clear impact on the chemical changes in ZPP.

### Non-isothermal DSC thermograms and heat of reaction

**Figure 3:**
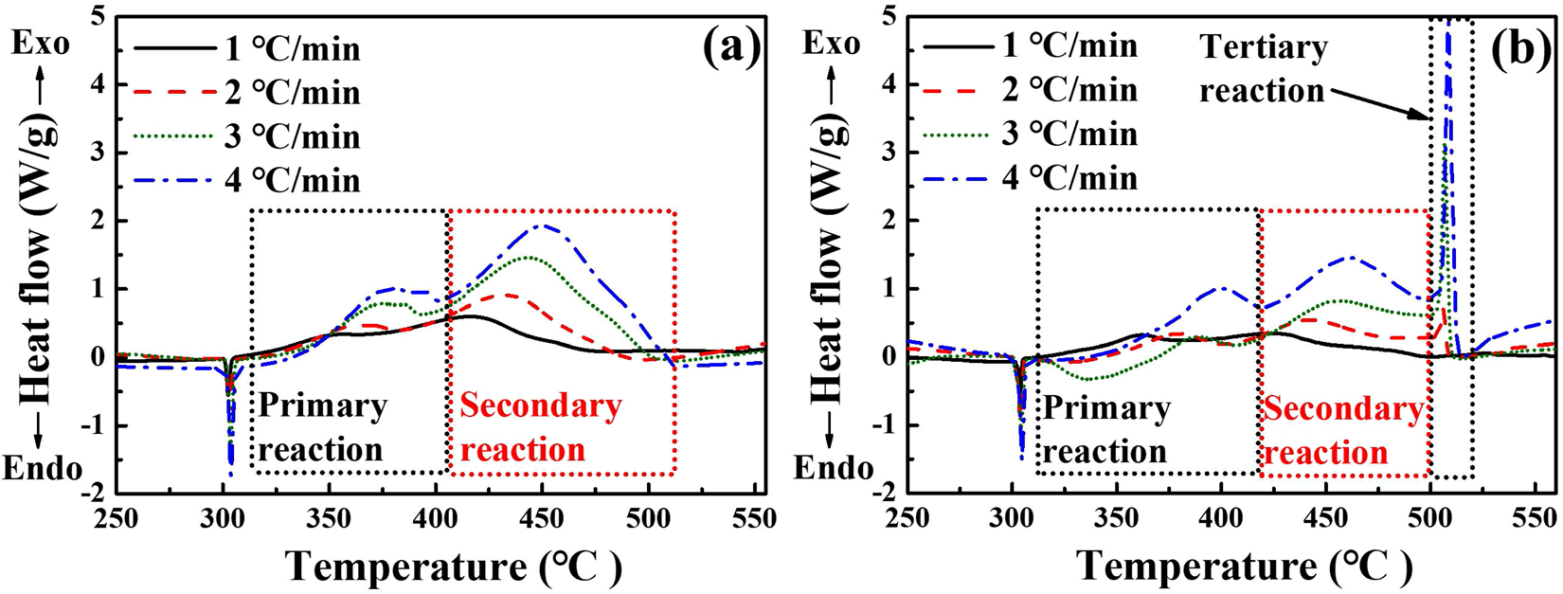
DSC thermograms for ZPP Samples (**a**) 0 and (**b**) 16. Sample 16, which was aged at 100% RH for 12 weeks, showed an additional tertiary reaction representing the decomposition of the remaining KClO_4_. This exothermic reaction may have been due to the fuel being oxidised by ageing.

Clear differences in the reaction process were observed between the unaged and aged ZPP samples. Figure 3(a) shows the DSC thermograms of the unaged ZPP sample (Sample 0), which consist of one endothermic peak and broad exothermic peaks, with multiple reactions overlapping. The endothermic peak around 300 °C represents the KClO_4_ phase transition^8^. In addition, multiple exothermic sub-reactions are superimposed on the overall exothermic reaction process. The primary exothermic peak, at around 320 °C–400 °C, can be attributed to the reaction between fuel, oxidants and Viton b, according to the relevant study^32^. Sanborn *et al*.^4^ reported that Viton b decomposes at around 400 °C with nearly 100% mass loss, indicating that the binder can initiate the exothermic reaction. The following exothermic peak can then be seen as a combustion reaction between the fuel and oxidants without Viton b^32^. Meanwhile, Fig. 3(b) shows the results for Sample 16, which was aged under a limiting humidity condition at 100% RH. This shows a new exothermic peak at around 500 °C due to the decomposition of the remaining KClO_4_^8^. This implies that the amount of ZrO_2_, which cannot act as a reducing agent, increased sharply because of hygrothermal ageing, leading to it the remaining KClO_4_ in Sample 16 decomposes.

DSC curves at a heating rate of 3 °C/min, showing how the peaks change with the ageing duration (see Supplementary Fig. S3). Only at 100% RH did the KClO_4_ decomposition reaction occur in all samples. However, the reaction did also occur in the 70% RH samples aged for at least 16 weeks, implying that sufficient ZrO_2_ can form at lower RH levels provided that the ageing duration is long enough.

The DSC thermograms were also used to calculate the heat of reaction *∆H* using Advanced Kinetics and Technology Solutions (AKTS) software^5^, utilising a sigmoidal tangential baseline to identify the initial and final heat flow changes. Table S1 and Fig. S4 in Supplementary information show the heat released by all ZPP samples, indicating that the heat of reaction decreased as the ageing duration increased. Even though the hygrothermally aged samples were only aged for a few weeks, their released heat values were similar to those of the thermally aged samples, and thus it is highly probable that the RH level caused a substantial decline in the heat released by the ZPP, as depicted in Fig. 1.

### Reaction kinetics based on isoconversional analyses

**Figure 4:**
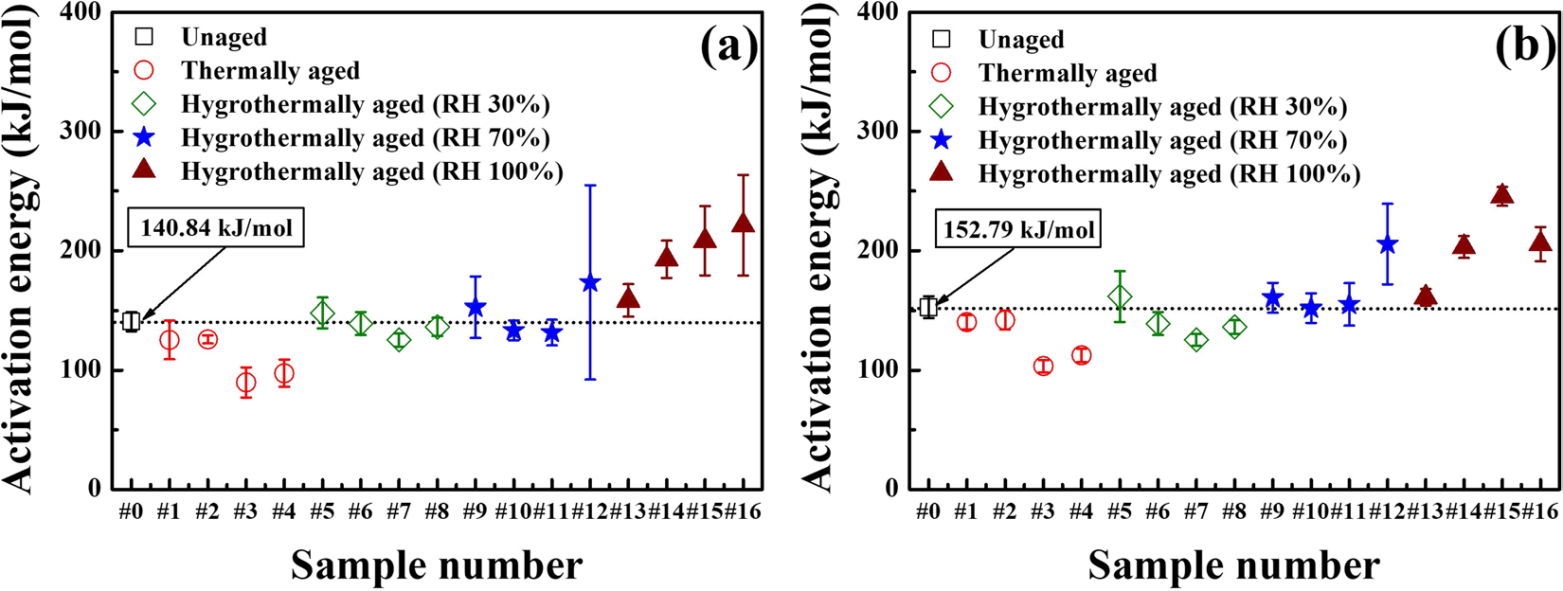
Activation energies for all ZPP samples, calculated using the (**a**) Friedman and (**b**) Ozawa methods. Both methods show similar trends among all samples. The activation energies are lower for the thermal ageing case but increase considerably under higher RH conditions. These results indicate that KClO_3_, an intermediate product of thermal ageing, can lower the activation energy. The high-RH results also indicate that the increases in ZrO_2_ content due to hygrothermal ageing made it more difficult for the fuel and oxidants to react.

The activation energy, *E*_*α*_ values were calculated using AKTS^5^ based on two different isoconversional methods (the Friedman^33^ and Ozawa^34^ methods), which adhere to the isoconversional principle^33–35^. The intrinsic error stemmed from high sensitivity to noise of Friedman method may give rise to a large deviation in the kinetic parameters. Thus, by employing Ozawa method which gives the average or integrated parameters of reaction, in conjunction with Friedman method, the error from Friedman alone can be checked and adjusted if necessary. Figure 4 shows the *E*_*α*_ values produced by the Friedman and Ozawa methods for all ZPP samples. In addition, Supplementary Table S2 shows the activation energy range, average value *E*_*aver*_, reference value *E*_*ref*_ and correlation coefficient *R*^2^ for both methods. Here, a difference can be observed between the experimental and reference *E*_*α*_ values because of the facts that the reference values were calculated for a restricted temperature range of 654.17–684.89 K and that they were obtained using the ASTM E698-99 method^36^. By contrast, in the current study, the *E*_*α*_ values are estimated by the Friedman and Ozawa methods.

In Fig. 4, the symbols represent the samples’ *E*_*aver*_ values with standard deviation. As the overall reaction process consists of multiple reactions, which can occur simultaneously, the *E*_*α*_ values fluctuate until the reaction terminates^37^. As a result, the maximum and minimum values can differ widely for different samples, and the extreme kinetic parameters of the reaction’s initial and final stages generally lead to high uncertainty. Thus, the current study was restricted to an *α* range of 0.2–0.8 to avoid such high parameter ambiguity, where *α* indicates the progress of reaction or the extent of conversion.

The trends in the *E*_*α*_ values for both methods are generally very similar, although there are some differences resulting from the calculation. These results appear to indicate that higher RH levels yield increased *E*_*α*_ values. Figure 4(a), obtained using the Friedman method, shows that the thermal ageing group (Samples 1–4) exhibited lower *E*_*α*_ values than Sample 0. This can be attributed to KClO_4_ decomposition creating KClO_3_ as an intermediate reaction product, which is well known to be generally more reactive and sensitive than KClO_4_^38–40^. This KClO_3_ can then facilitate the exothermic oxidiser–fuel reaction, causing the lower *E*_*α*_ needed to complete the overall thermal reaction. Under hygrothermal ageing conditions, *E*_*α*_ increased with the RH level, which is assumed to be due to the increased moisture content leading to Zr oxidation, which can yield unreacted KClO_4_. Moreover, ZrO_2_ is quite stable, making ZPP combustion more difficult. Thus, high-RH levels result in an increased *E*_*α*_, causing the ZPP to become less reactive.

The ageing duration also influenced the ZPP ageing process: longer ageing produced ZPP samples with more widely varying *E*_*α*_–*α* relationships. These large kinetic parameter variations confirm that the overall exothermic reaction is composed of multiple simultaneous reactions^38,41^, indicating that the reaction process becomes more complex as the material ages. On the other hand, sample #12 corresponds to 16 weeks of hygrothermal ageing at 70% RH, which is the longest accelerated ageing duration. It is presumed that the inhomogeneity of the sample composition and deviation of the particle size distribution have attributed to the larger variation of the activation energy. The *E*_*α*_ values obtained using the Ozawa method (Fig. 4(b)) showed similar trends to the Friedman method results, although the correlation coefficient *R*^2^ (See Supplementary Table S2) was slightly higher. The similarity of the *E*_*α*_ values obtained by both methods implies that the trends that they show are reasonably accurate.

### Peak deconvolution analyses

The overall thermal reaction is composed of various overlapping thermal sub-reactions, and its properties may therefore change depending on the dominant reaction. To investigate the ageing effect in more detail, peak deconvolution analyses were also carried out. On the basis of previous works, four sub-reactions are believed to make up the overall ZPP reaction^8,32,42^, namely combustion reactions among Viton b, Zr, KClO_3_ and KClO_4_ (Peak 1), combustion reactions among Zr, KClO_3_ and KClO_4_ (Peak 2), decomposition of pure KClO_3_ (Peak 3) and decomposition of pure KClO_4_ (Peak 4). The representative of overall reactions can be found as Supplementary Fig. S5. In addition, KCl and ZrO_2_ were not involved in the deconvolution analyses because KCl decomposes at about 770 °C and ZrO_2_ maintains its chemical structure until at least 1100°, well beyond the temperature range of the current study. The peak deconvolution results are based on the DSC data taken at 3 °C/min.

**Figure 5:**
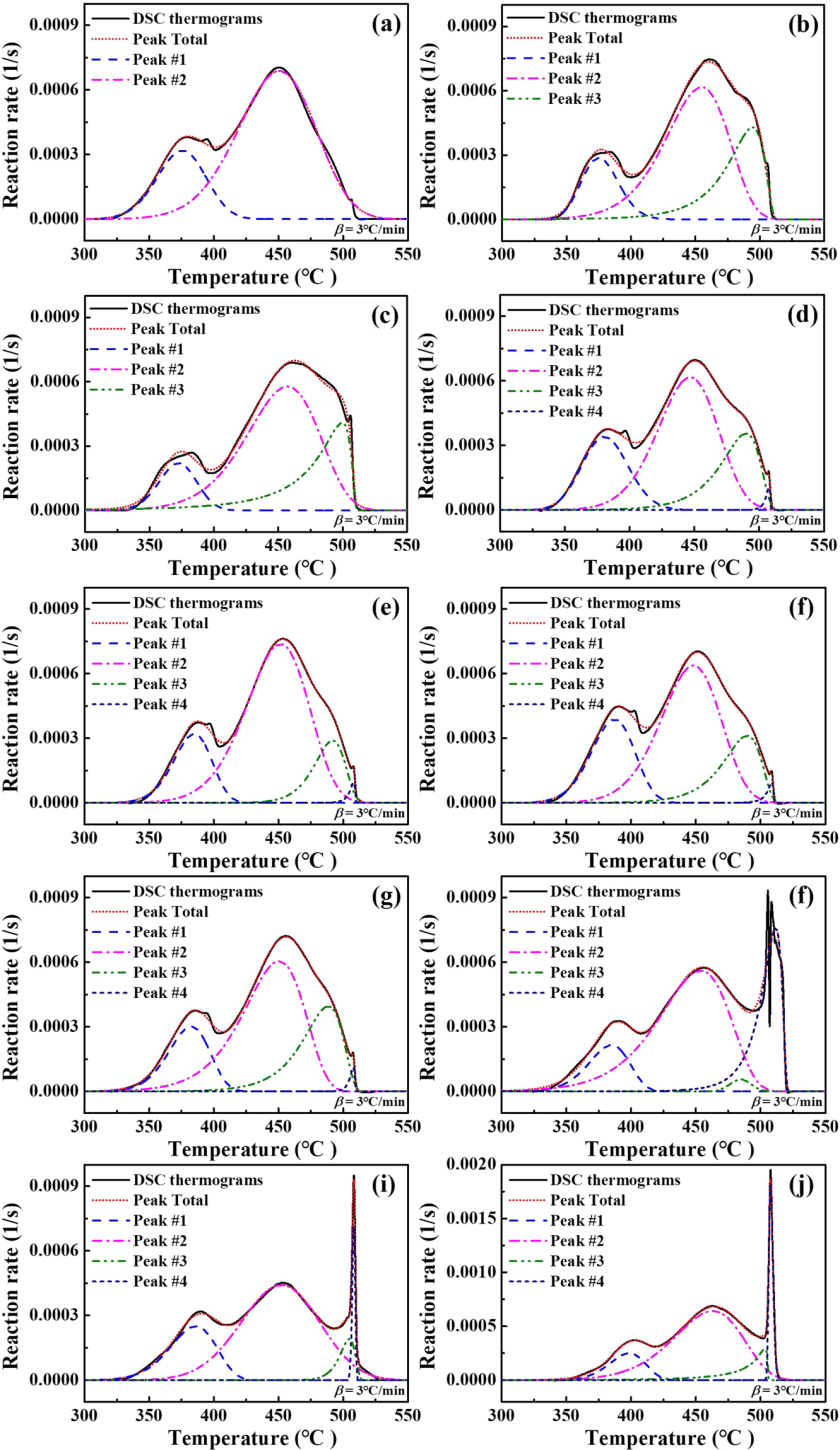
Peak deconvolution results, showing the reaction rates in the thermal and hygrothermal ageing cases, namely Samples (**a**) 0, (**b**) 1, (**c**) 4, (**d**) 5, (**e**) 7, (**f**) 9, (**g**) 11, (**h**) 13, (**i**) 14, and (**j**) 16. In the thermal ageing process, KClO_3_ decomposition (Peak 3) is shown as the most significant reaction. In the hygrothermal ageing process, these results indicate that the exothermic reaction of the unreacted KClO_4_ (Peak 4) is the most significant reaction.

Figure 5 shows the reaction rates for unaged (Fig. 5(a)), thermal (Fig. 5(b,c)), and hygrothermal ageing cases (Fig. 5(d–j)). The thermally aged ZPP samples, which generally include the three exothermic reactions (Peaks 1, 2 and 3). Of these reactions, KClO_3_ decomposition (Peak 3) appears to be the most significant reaction in the thermal ageing process, in accordance with the XPS analyses (Fig. 2(a)), which show a substantial KClO_3_ content.

As shown in Fig. 5(d–j), the hygrothermal ageing process includes all four thermal reactions. Figure 5(d–g), representing the 30% and 70% RH samples, are apparently identical to thermal results, and those for the 100% RH samples are notably different (Fig. 5(h–j)). Here, Peak 4 is particularly pronounced. In addition, the XPS study also showed that the KClO_3_ content decreased over time, causing the low KClO_3_ decomposition rate to be seen in Fig. 5(h–j).

Peak 1, corresponding to the exothermic reaction with the binder, also shows the reaction rate decreasing over time in both ageing cases. Meanwhile, referring to the quantitative data based on XPS results, the decreasing trend of the amount of Viton b caused by ageing can be found in Supplementary Fig. S6. These phenomena can also explain the weaker coherence between the fuel and oxidants and may have led to the performance degradation and changes in *E*_*α*_. Figure 1 represents the binder degradation process by the increasing spaces between binder molecules.

### Simulation of the ZPP reaction progress using the extracted kinetics

**Figure 6:**
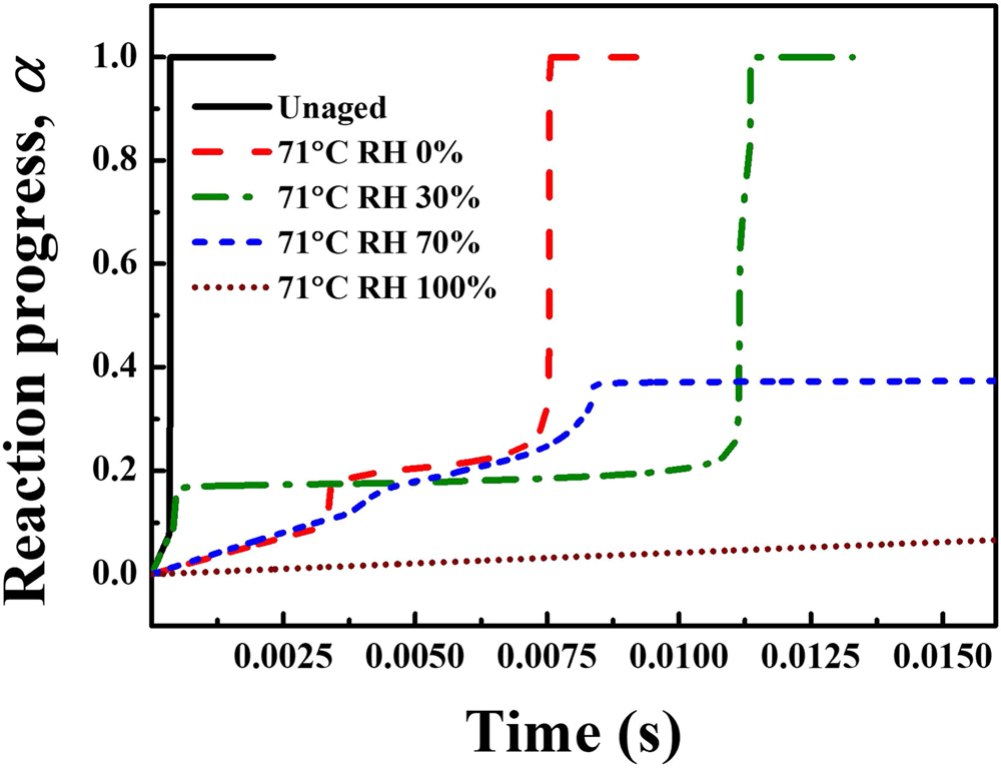
Calculated reaction of each representative ZPP samples from five sample groups of unaged, thermally aged, and hygrothermally aged. Based on the Arrhenius kinetics parameters extracted from DSC, aged samples with severe RH conditions tend to show incomplete burning and significant delay in the ignition.

Figure 6 shows the extent of conversion from unreacted (*α* = 0) to fully reacted (*α* = 1). The activation energy *E*_*α*_ and pre-exponential factor *A*_*α*_ are obtained from DSC experiments for each corresponding *α*. The Arrhenius equation which is solved for unaged sample and four different aged samples is as follows,

1 $$\frac{d\alpha }{dt}={A}_{\alpha }\exp (-\frac{{E}_{\alpha }}{R{T}_{\alpha }})$$

where the universal gas constant is *R*, temperature *T*_*α*_ is in Kelvin, the activation energy is in kJ mol^−1^, and pre-exponential factor at the corresponding progress of reaction is in s^−1^.

In this simulation of constant volume reaction for ZPP, the density and specific heat at constant pressure are taken as 4600 kg/m^3^ and 250 J/kg-K, respectively^43^. The samples are ensured to ignite instantly at a preheated value of 1100 K initially, and watched to progress into a full reaction. As shown, the rate of reaction progress is delayed with each additional aging while high humidity conditions gave rise to a misfire or incomplete reaction at about 35% conversion for RH 70% case. For the present initial condition, RH 100% sample failed to react.

### Predicted lifespan

**Figure 7:**
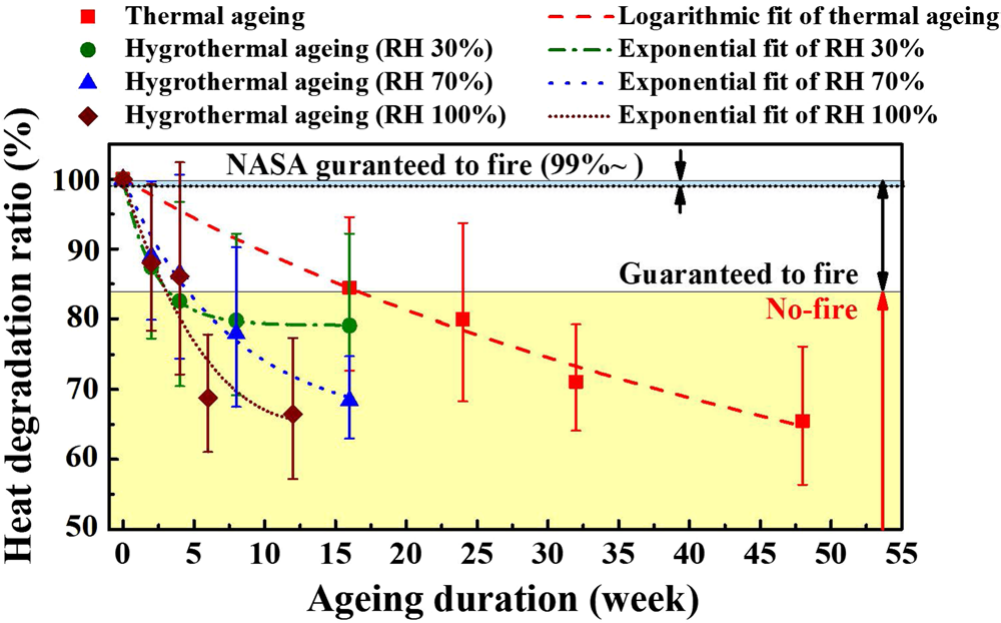
The heat degradation ratio (*∆H*_*aged*_ */∆H*_*unaged*_) of all aged ZPP samples and the guaranteed to fire limit for estimating the ZPP lifespan. The heats of reaction for thermally aged ZPP followed a logarithmical decay while the enthalpies for hygrothermally aged samples degraded exponentially. Thus, it can be suggested that the humidity accelerates the heat degradation. Meanwhile, the lifespans were estimated by utilising both the experimental data within the ‘guaranteed-to-fire’ limit and the van’t Hoff equation.

Although ZPP is a fairly stable pyrotechnic compound, the prediction of its lifespan related to various ageing conditions can be useful. Figure 7 demonstrates the pattern of how reaction enthalpy is reduced for each ZPP sample at the varying ageing duration. In particular, the heat degradation ratio indicates how aged samples change per unaged sample in terms of their enthalpy of reaction (${\rm{\Delta }}{H}_{aged}/{\rm{\Delta }}{H}_{unaged}$). Prior to referring to a lifespan of each sample, the different patterns of heat of reaction decay were observed. The thermal ageing pattern followed a logarithmic decay whereas the hygrothermal ageing cases were well fitted with exponential curves that showed a rapid degradation of heat performance. Under the increased RH conditions, the heat of reaction decreased even significantly. The utilised empirical fits are as follows:

2 $$\frac{{\rm{\Delta }}{H}_{aged}}{{\rm{\Delta }}{H}_{unaged}}={\phi }_{1}{e}^{{\phi }_{2}t}+{\phi }_{0}\,({\rm{exponential}}\,{\rm{fit}}\,1)$$

3 $$\frac{{\rm{\Delta }}{H}_{aged}}{{\rm{\Delta }}{H}_{unaged}}={e}^{{\phi }_{0}+{\phi }_{1}t+{\phi }_{2}{t}^{2}}\,({\rm{exponential}}\,{\rm{fit}}\,2)$$

4 $$\frac{{\rm{\Delta }}{H}_{aged}}{{\rm{\Delta }}{H}_{unaged}}=\,\mathrm{ln}({\phi }_{0}+{\phi }_{1}t)\,({\rm{logarithmic}}\,{\rm{fit}})$$

Here, *t* is the ageing durations (in weeks), and *φ*_0_, *φ*_1_ and *φ*_2_ are constant coefficients. In this study, the empirical model with the smallest root mean square error was selected, that is, the fit whose root squared error *r*^2^ is closest to 1. The utilised parameters are provided in Supplementary Table S3.

Although pyrotechnic initiators are designed to produce at least twice the required heat release^44^, degraded performances may further occur by the various operational and environmental conditions, besides the temperature and RH conditions. For instance, Burgess *et al*.^45^ have reported that exposing to UV radiation can cause the formation of radicals and some portion of impurities by absorbing light for explosives or polymers. Also, Thompson *et al*.^46^ have observed the changes in the physical properties and performance of explosives under the thermal cycling tests.

Thus, a limit, guaranteed to fire of ZPP as shown in Fig. 7, was set to a value higher than 84% of ${\rm{\Delta }}{H}_{unaged}$, which was the highest reaction enthalpy obtained in the samples. Utilising the experimental data within a ‘guaranteed to fire’ limit in Fig. 7, the lifespan is estimated by substituting the *AF*, obtained from the Arrhenius equation, into the van’t Hoff equation. Both Friedman and Ozawa are used as the basis of isoconversional methods of Table 2. In case of Friedman, the corresponding lifespan for unaged ZPP sample was predicted to be 277.48 years. The four-month thermally-aged ZPP indicates that the sample can be utilised for about 120 years. Moreover, all three hygrothermal samples showed even shorter lifespan in comparison to thermal-only ageing condition, suggesting that humidity is responsible for the shortened lifespan. The lifespan for Ozawa method showed the identical lifespan trend with Friedman, as well. Table 2 summarizes the lifespans and parameters utilised in calculating the lifespan.

**Table 2:** Lifespan comparison of unaged and aged samples and acceleration factors.

Sample label	Ageing condition	Acceleration factor		Lifespan (year)	
		Friedman	Ozawa	Friedman	Ozawa
0	Unaged	4.18	4.72	277.48	485.15
1	0% RH 71 °C	3.58	4.16	117.87	124.27
4	30% RH 71 °C	5.09	5.17	68.15	73.41
9	70% RH 71 °C	4.72	5.11	48.49	70.98
11	100% RH 71 °C	4.60	5.08	43.04	68.12

Also, the recommended usage time of off-the-shelf ZPP cartridge is provided to about 10 years^47^, which corresponds to a calculated lifespan for unaged ZPP until preserving its heat value of 99%. Thus, the range of NASA guaranteed to fire was set to more than 99% of enthalpy of unaged ZPP represented in Fig. 7. The provided 10 years of the usage time may be the value satisfying the constraint for the end product of maximum reliability.

## Discussion

One of the concerning issues associated with ageing of pyrotechnic substances is the metal fuel oxidising as it is exposed to the atmosphere during the mixing and loading processes, subsequently shortening the shelf life. The extent of Zr oxidisation and oxidants were quantified by analysing the XPS spectra and clarified by analysing the slight *E*_*α*_ variations exhibited by the isoconversional methods. In general, oxidisation of the fuel increases the *E*_*α*_ value because the fuel’s oxidised shell inhibits its reaction with the oxidant, though this only occurred under the high-RH condition in the present study. In most cases, *E*_*α*_ either remains the same or decreases over time due to the combined effects between oxidised fuel and decomposed oxidants. Hence, the anomalous change in *E*_*α*_ suggests that the behaviour of the combustion reaction of ZPP is highly complex. Also, the ageing of ZPP revealed its affects in the DSC thermograms. Applying deconvolution to the DSC thermograms showed that ZPP oxidisation may generate off-stoichiometric compositions that lead to performance degradation and rather more complex reaction mechanisms than the simple combustion reaction seen in the unaged sample. Overall results may suggest the performance degradation in the end.

The aged ZPP samples’ heats of reaction decreased substantially over time and with increasing RH. This change was presumably due to Zr oxidisation, reduced oxidant levels, and the aged samples’ off-stoichiometric compositions. Remarkably, high-RH condition resulted in the considerably low heats of reaction. Although it could be argued that the ageing analyses should include the heats of reaction for ZrO_2_, KCl, and any new products, in order to better assess the effect of ageing on the heat values of ZPP initiators, these make up a very small part, compared with the amount of ZPP. Thus, they are unlikely to have a significant impact on the ZPP ageing results.

The aged ZPP samples’ lifespans were also estimated by utilising the van’t Hoff equation on the basis of the activation energy values obtained from Friedman and Ozawa methods. The ZPP aged under high-RH conditions had substantially decreased lifespan. Moreover, lifespan can also be affected by a variety of other factors such as vibration, shock, or thermal cycling, in addition to the temperature and humidity, which reflects that the actual lifespan of ZPP is likely to be shorter than these estimates, causing a misfire or failure during reaction progress. Thus, the storing condition of ZPP must remain ‘dry’ to retain its intended energetic performance.

Although how one can relate the present accelerated aging samples with the naturally aged samples is remained open for debate, this study has provided useful insight into the effects of temperature and humidity on ageing, including the main trends, changes in composition and oxidisation levels, combustion characteristics, and expected lifespan under different storage conditions.

## Methods

### Materials

The ZPP materials used in the present study were in powder form, sifted through a 200 mesh (75 μm) particle sieve. They were composed of 52 wt% Zr (Rockwood Lithium, ~2 μm, USA), 42 wt% KClO_4_ (Barium & Chemical Inc., ~6 μm, USA), 5 wt% Viton b ([-C_7_H_2_F_12_-]_*n*_) (Dupont, USA)^48^ and 1 wt% graphite.

Prior to the DSC experiments, the ZPP samples were aged at 71 °C at several different RH levels (30%, 70% and 100%) for different periods of time. The selected ageing temperature (71 °C) follows aerospace industry guidelines^44^, and the RH conditions (30–100%) were chosen so as to investigate RH-related trends and effects on the ZPP mixtures. The ZPP samples were divided into three different ageing groups: an unaged sample, plus thermal and hygrothermal ageing. The sample conditions are summarised in Table 1, and throughout this paper, the samples are discussed using the sample numbers listed there.

It should be noted, however, that the ageing durations used for the two ageing groups (thermal and hygrothermal) were not the same. As shown in Table 1, the thermal ageing group samples (1–4) were aged much longer than the hygrothermal ageing group samples (5–16). In addition, the ageing times for the hygrothermal ageing samples in the 100% RH case (13–16) were different from those used for the other cases (5–12). Although this means that an accurate quantitative comparison between the thermal and hygrothermal ageing groups would be challenging, it still enabled the effect of thermal ageing on ZPP materials to be observed, as well as approximate comparisons to be made regarding the effect of different RH levels.

### XPS study

The ZPP mixtures’ chemical bonding states, relative composition and detailed XPS spectra were measured using an ultra-high-vacuum (UHV) AXIS-SUPRA photoelectron spectrometer (Kratos, UK). This was equipped with micro-focused monochromatised Al Kα X-ray sources (1486.6 eV) and a hemispherical analyser (WX-600). The base pressures in the sample analysis and load lock chambers were less than 5 × 10^−10^ and 5 × 10^−8^ Torr, respectively.

### DSC study

Calorimetry experiments were also conducted to investigate the ZPP mixtures’ thermodynamic characteristics and thermal reaction processes. To ensure that the kinetic analyses were accurate, the DSC data were collected following the International Confederation for Thermal Analysis and Calorimetry (ICTAC) Kinetics Committee’s recommendations^49^. The DSC experiments were performed using a DSC-3 SCcalorimeter (Mettler Toledo) in an 85 mL/min nitrogen atmosphere at heating rates of 1, 2, 3 and 4 °C/min over a temperature range of 30 °C–600 °C. These slow heating rates (up to 4 °C/min) were adopted so as to observe the various thermodynamic reaction processes clearly^15^.

When conducting DSC experiments, the sample masses can affect the calorimetry results^49^: if the sample mass is too high and the heating rate is too fast, self-heating may occur during the exothermic reactions, leading to inaccurate results^36,50^. On the other hand, if the sample is too light, the exothermic peak in the thermograms may be too small to distinguish from the other noise peaks. To avoid this issue, sample masses of 2–3 mg were found to be appropriate on the basis of repeated experiments.

Most samples showed good repeatability with regard to the DSC thermograms while there were some variations on several samples associated with the errors from sample weighing, machine accuracy of sensors, and sample uniformity. Thus, DSC experiments were repeated multiple times for obtaining the suitable thermograms without any peculiar variations or noise. The current study has provided the representative figure (see Supplementary Fig. S7) showing the two cases: the first one showing good repeatability for all three trials and the second one showing variations for the first trial and stabilized afterwards. The ZPP mixtures were placed in a standard 40 μL pierced aluminium pan, distributed in a thin, even layer over the bottom of the pan to prevent focusing the heat in one place.

### Isoconversional-method-based reaction kinetics calculations

The kinetic triplet, which comprises the activation energy *E*_*α*_, pre-exponential factor *A* and reaction model *f* (*α*), plays a key role in determining the reaction kinetics^35^. In this study, the kinetic parameters were calculated using AKTS software^50^. The values of the activation energy *E*_*α*_ were obtained using two different and widely used isoconversional methods, namely the Friedman^33^ and Ozawa^34^ methods. Although both methods determine the instantaneous activation energy per varying progress of reaction *α*, the Friedman method provides the kinetic parameters based on the differential Arrhenius equation

5 $$\mathrm{ln}[{({\beta }_{i}\frac{d\alpha }{dt})}_{\alpha ,i}]=\,\mathrm{ln}[f(\alpha ){A}_{\alpha }]-\frac{{E}_{\alpha }}{R{T}_{\alpha ,i}},$$

while the Ozawa method uses the integral form of Arrhenius equation

6 $$\mathrm{ln}({\beta }_{i})=const-1.052(\frac{{E}_{\alpha }}{R{T}_{\alpha }}),$$

where the subscript *i* represents the DSC experiment number (carried out at a heating rate of *β*_*i*_) and *α* denotes the extent of conversion^35^. In addition, *R*, *T*_*α*_, *E*_*α*_, *A*_*α*_ and *f* (*α*) are the universal gas constant, temperature (in K), activation energy (in kJ mol^−1^), pre-exponential factor at the corresponding *α* (in s^−1^) and reaction model, respectively^35^. Since the activation energy was obtained based on non-isothermal DSC experiments in this study, these Arrhenius equations are also for the non-isothermal case. To compare the changes in activation energy under different ageing conditions and obtain more accurate values for each step of the reaction, values were calculated using both isoconversional methods.

### Peak deconvolution method

AKTS^51^ was also used to separate the overlapping reaction peaks via deconvolution using the Fraser–Suzuki function^52,53^. Peak deconvolution was applied to the reaction rate–temperature relationships seen in the ZPP samples heated at 3 °C/min, obtained from the kinetic analyses described above.

### Lifespan calculation

The aged ZPP samples’ lifespans were calculated using the van’t Hoff equation, which is commonly used to obtain the usage time of solid propellants subjected to the accelerated ageing. The equation is particularly suitable for ageing that yields the changes in chemical composition, such that

7 $${t}_{E}={t}_{T}\cdot A{F}^{\frac{({T}_{T}-{T}_{E})}{{\rm{\Delta }}{T}_{F}}}/365.25,$$

where *t*_*E*_ is the estimated lifespan (in years) at a storage temperature of *T*_*E*_ (in °C), *t*_*T*_ is the ageing duration (in days) at an ageing temperature of *T*_*T*_ (in °C) and *AF* is the factor by which the reaction rate changes for every *∆T*_*F*_ change in temperature. In this study, the *T*_*E*_ and *∆T*_*F*_ values were 25 °C and 10 °C, respectively. This equation was used to determine the relationship between the lifespan and heat degradation ratio.

The factor *AF* can be calculated indirectly from the Arrhenius equation and can be expressed as

8 $$AF=\exp (+\frac{{E}_{\alpha }}{R}\cdot \frac{{\rm{\Delta }}{T}_{F}}{{T}_{T}^{2}}),$$

The most appropriate range for this factor is generally 2–4^9,54^, so the parameters used in the study were chosen such that the data yielded *AF* values in this range and were highly correlated for calculating ZPP lifespans accurately.

## Supplementary information

Supplementary file
