# Supplementary material for: Towards understanding the effects of heat and humidity on ageing of a NASA standard pyrotechnic igniter
Source: Sci Rep. 2019 Jul 15;9:10203. doi: 10.1038/s41598-019-46608-8 (PMC6629647; doi:10.1038/s41598-019-46608-8)
Supplement: Supplementary file 1 — Supplementary file [file 41598_2019_46608_MOESM1_ESM.docx]

Electronic Supplementary Information

*For*

**Towards understanding the effects of heat and humidity on ageing of a NASA standard pyrotechnic igniter**

Juyoung Oh,^1^ Seung-gyo Jang,^2^ and Jack J. Yoh^1^*

*^1^Department of Mechanical and Aerospace Engineering, Seoul National University, Seoul, 08826, South Korea*

*^2^Agency for Defense Development, Daejeon, 34060, South Korea*

* Corresponding author:

*[jjyoh@snu.ac.kr](mailto:jjyoh@snu.ac.kr)*

**
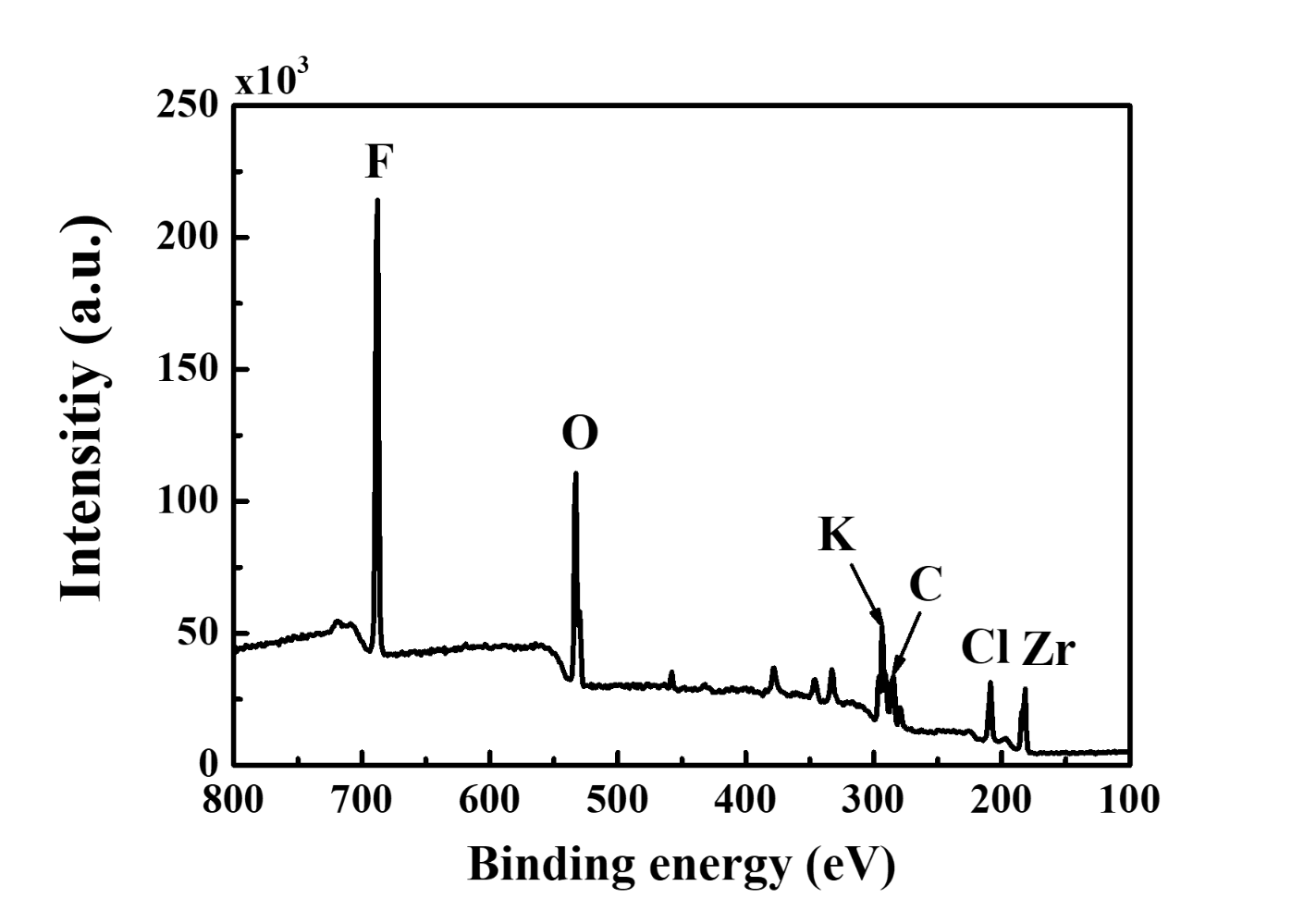
**

**Figure S1.** The ZPP composition obtained from XPS qualitative analysis.


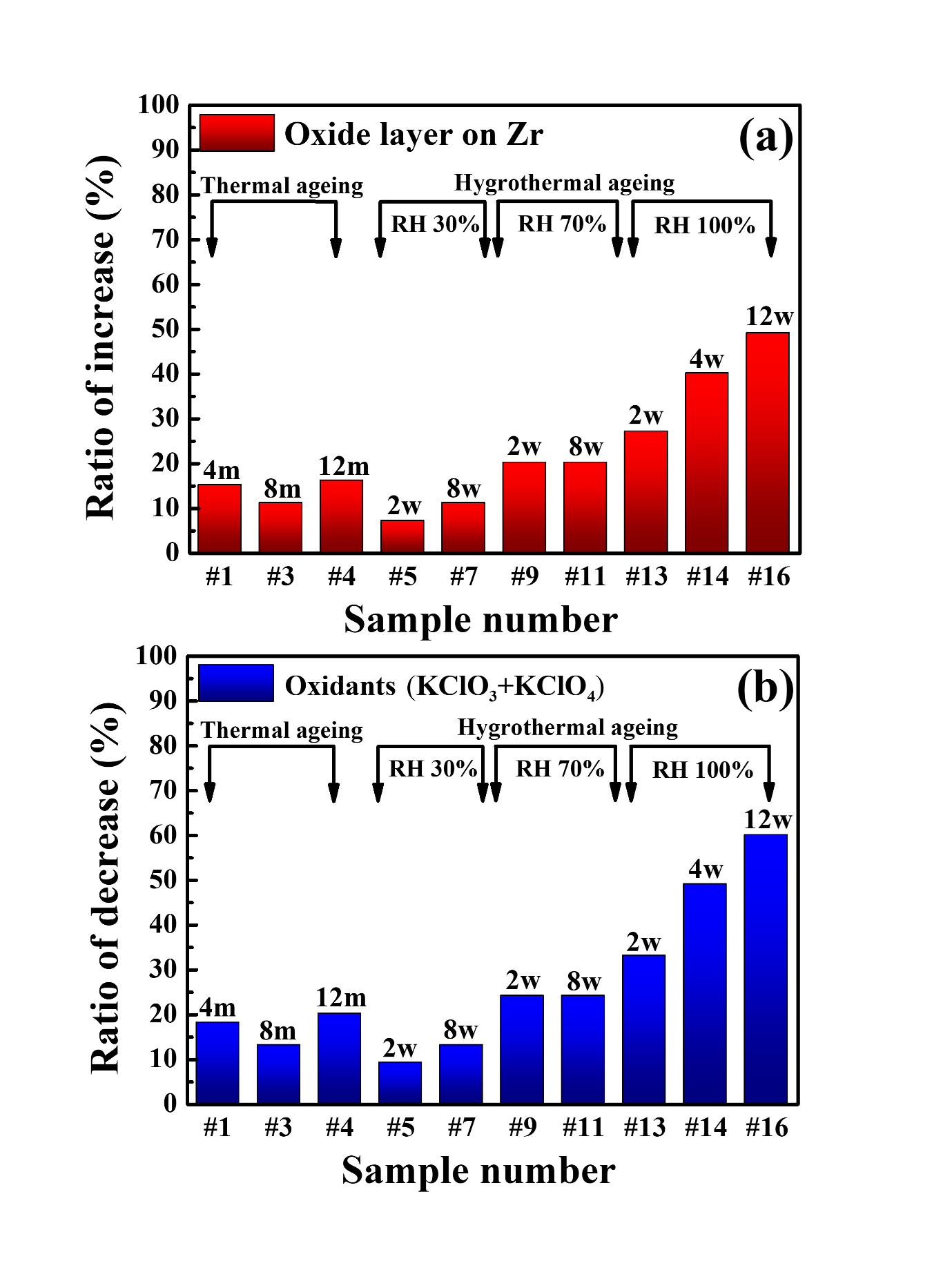


**Figure S2.** (a) Variation in oxide layer on Zr with various ageing type (b) Variation in oxidants content in ZPP with various ageing type. Zirconium oxidization tended to increase as both RH and ageing duration were increased while oxidants showed the decomposed trend.


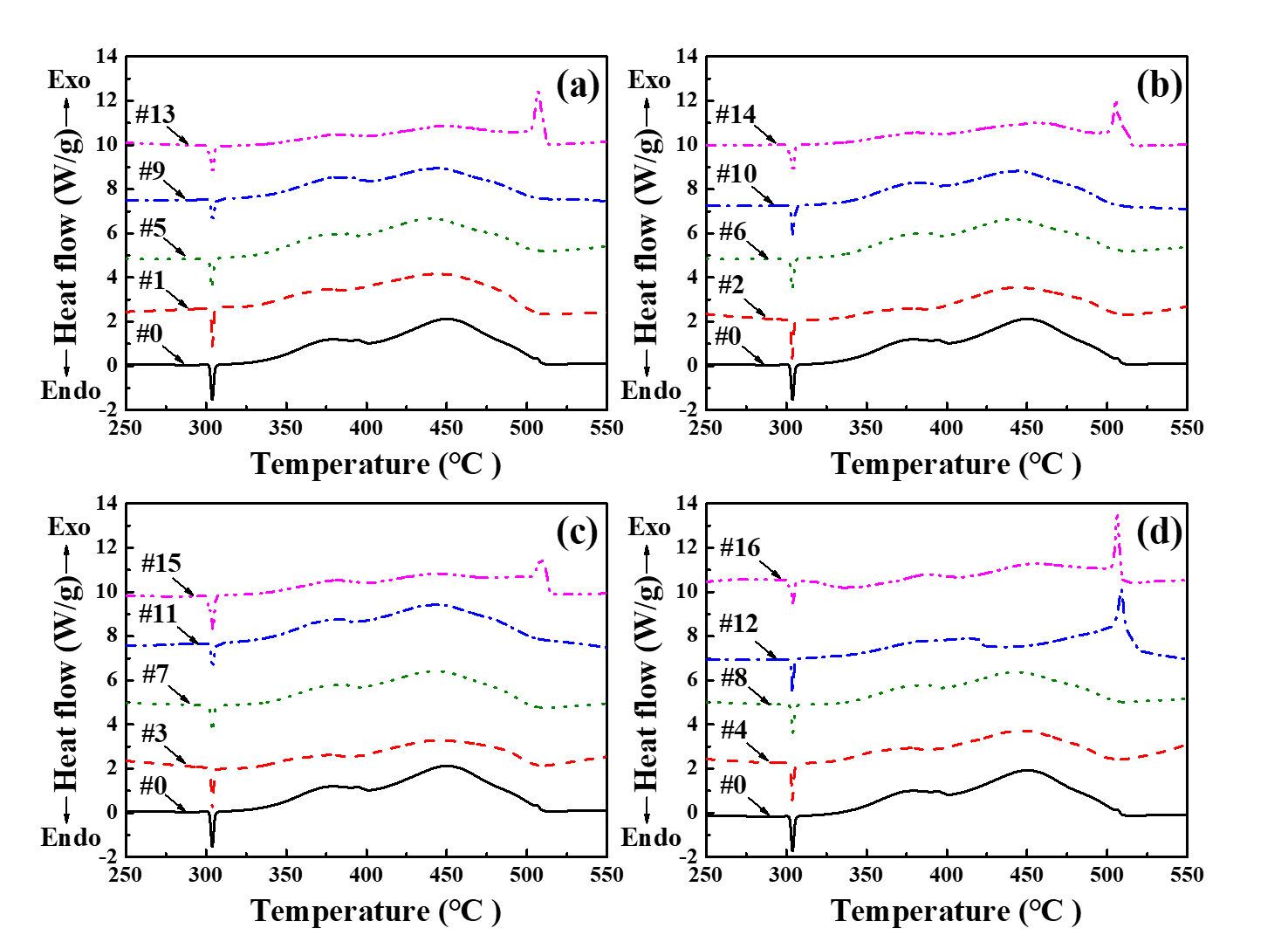


**Figure S3.** DSC thermograms (vertically offset) at a heating rate of 3°C/min for the samples aged for (a) 2, (b) 4, (c) 8 and (d) 16 weeks. Each DSC thermogram represents the result performed on the sample number indicated by an arrow. Higher thermogram positions indicate ZPP samples aged at higher RH levels. Only the 100% RH samples showed KClO_4_ decomposition reactions for all ageing durations. However, the sample aged for 16 weeks at 70% RH condition also showed a KClO_4_ decomposition peak, so both the ageing period and RH level can affect Zr oxidisation.


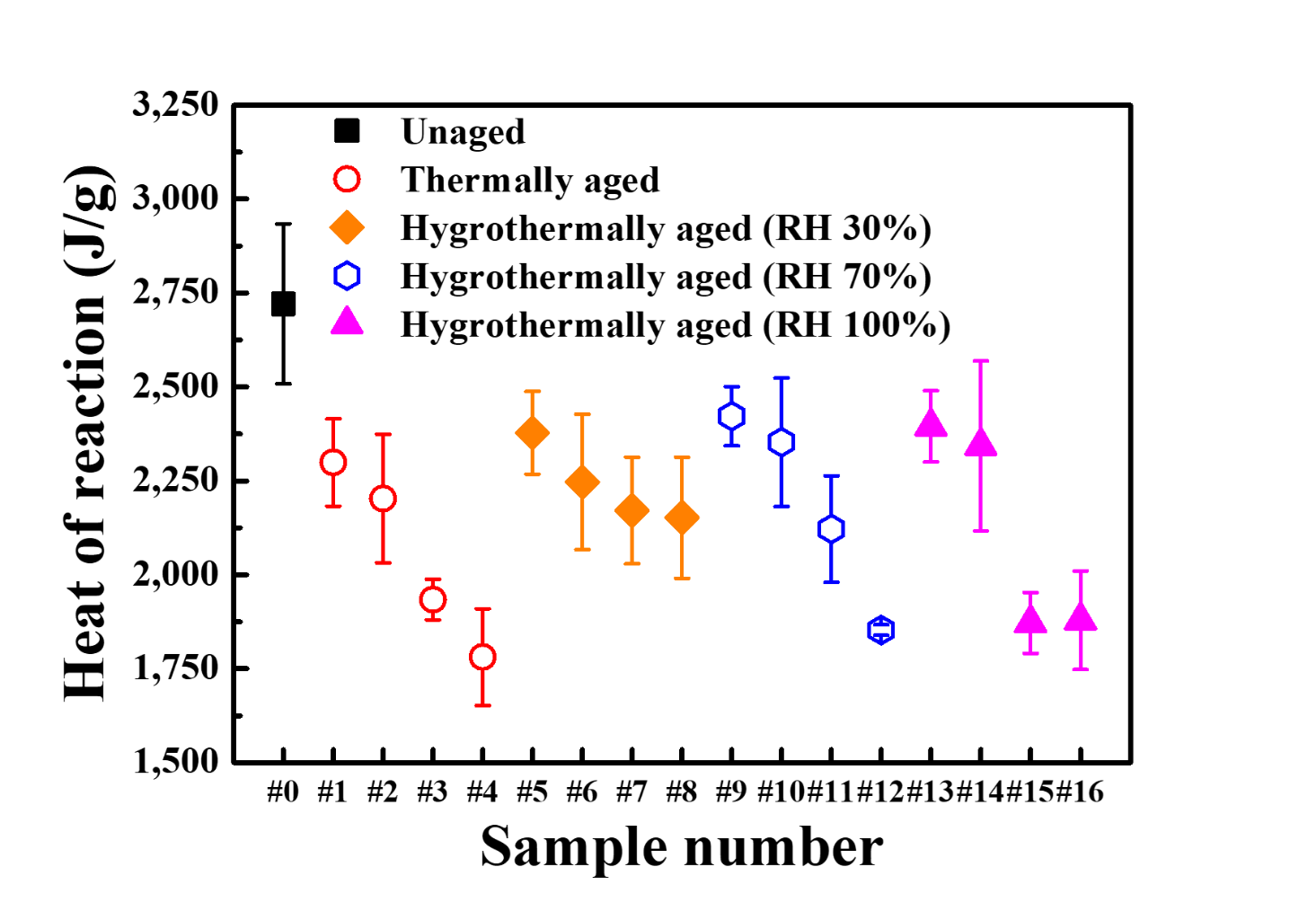


**Figure S4.** Heat of reaction values for all ZPP samples.


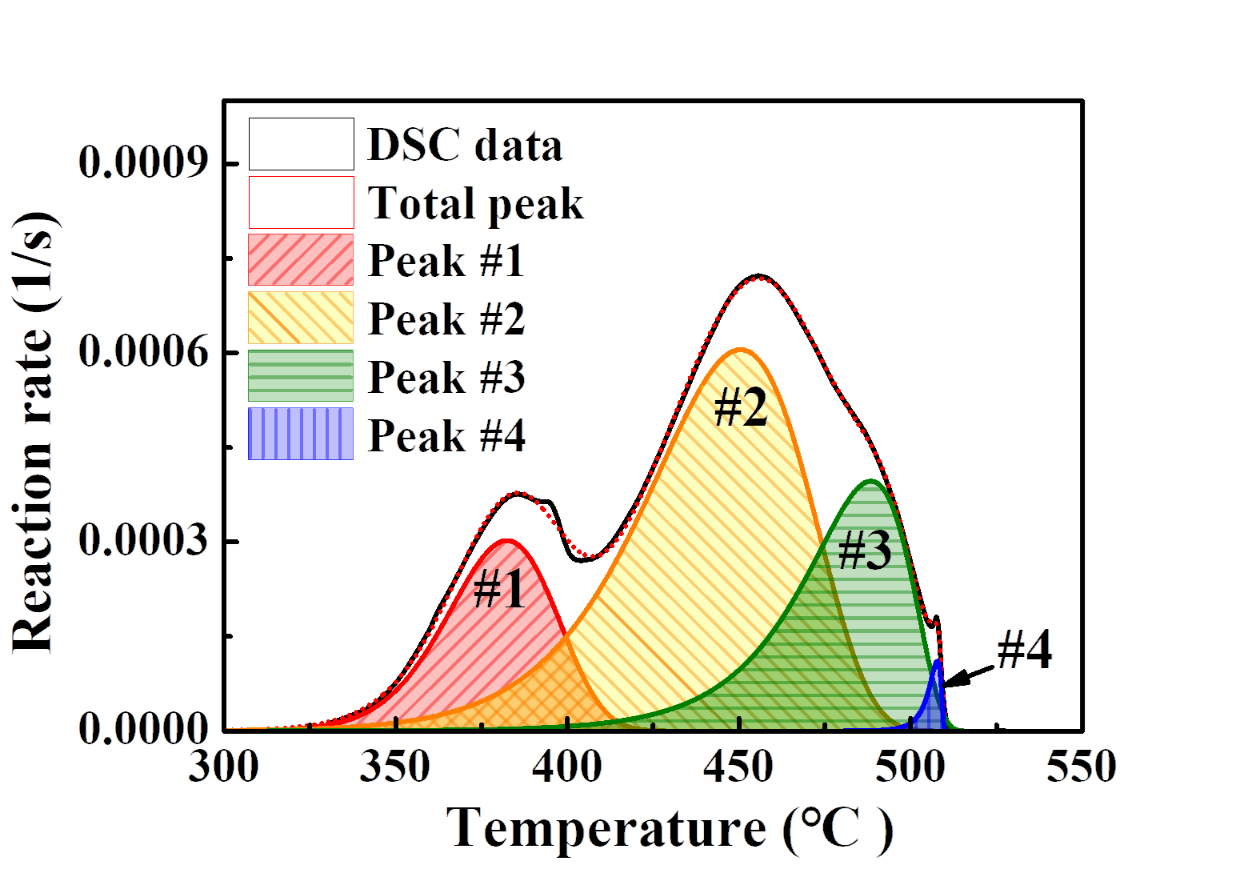


**Figure S5.** A representative of composition in ZPP peak applied to the deconvolution method.

**
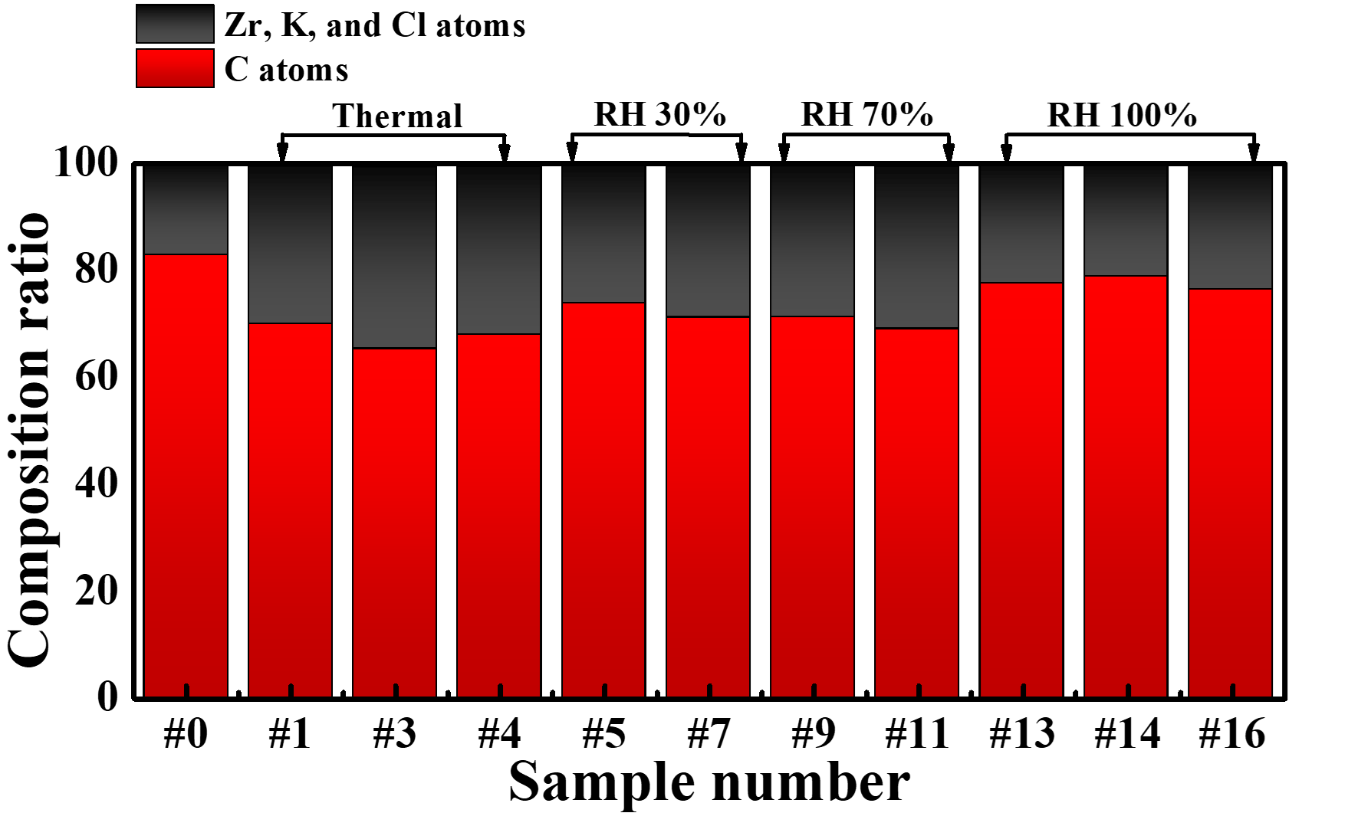
**

**Figure S6.** A ZPP composition ratio for each aged sample. ZPP composition ratio shown as two parts, namely the Viton b (C atoms) and the rest (fuel and oxidants). Viton b tended to decrease with the increasing ageing duration.


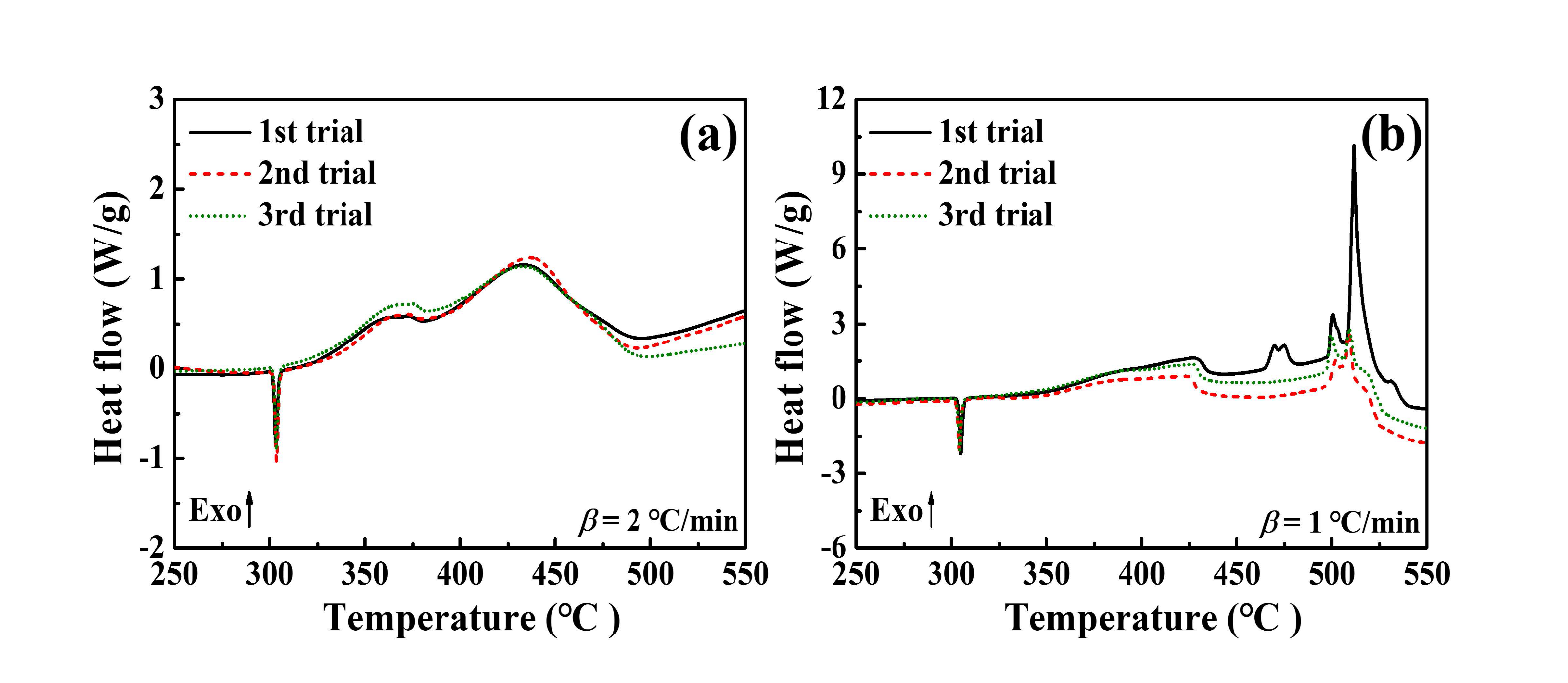


**Figure S7.** Repeatability of the DSC experiments for ZPP samples. (a) DSC thermograms of sample #0 at 2℃/min of heating rate showing good repeatability for all three trials and (b) DSC thermograms of sample #8 at 1℃/min of heating rate showing variations for the first trial and stabilized afterwards.

| Sample | *∆H* (J/g) | Ageing duration | Ageing type |
| --- | --- | --- | --- |
| 0 | 2721 $\pm$213 | - | Unaged |
| 1 | 2299 $\pm$ 116 | 4 months | Thermal ageing |
| 2 | 2203 $\pm$ 171 | 6 months |  |
| 3 | 1934 $\pm$ 54 | 8 months |  |
| 4 | 1781 $\pm$ 129 | 12 months |  |
| 5 | 2378 $\pm$ 110 | 2 weeks | Hygrothermal ageing  (30% RH) |
| 6 | 2247 $\pm$ 180 | 4 weeks |  |
| 7 | 2171 $\pm$ 142 | 8 weeks |  |
| 8 | 2152 $\pm$ 161 | 16 weeks |  |
| 9 | 2422 $\pm$ 79 | 2 weeks | Hygrothermal ageing  (70% RH) |
| 10 | 2353 $\pm$ 171 | 4 weeks |  |
| 11 | 2122 $\pm$ 142 | 8 weeks |  |
| 12 | 1853 $\pm$ 14 | 16 weeks |  |
| 13 | 2395 $\pm$ 95 | 2 weeks | Hygrothermal ageing  (100% RH) |
| 14 | 2343 $\pm$ 226 | 4 weeks |  |
| 15 | 1872 $\pm$ 81 | 6 weeks |  |
| 16 | 1809 $\pm$ 131 | 12 weeks |  |

**Table S1.** Heats of reaction for all samples.

| Sample | *E_α_* range (kJ/mol) | *E_aver_* (kJ/mol) | *E_ref_*  (kJ/mol) | *R^2^* | Ageing type |
| --- | --- | --- | --- | --- | --- |
| Friedman method | | | | | |
| 0 | 130–170 | 140.84 | 212.52^36^ | 0.9963 | Unaged |
| 1 | 91–148 | 125.47 |  | 0.9967 | Thermal ageing |
| 2 | 120–134 | 125.88 |  | 0.9890 |  |
| 3 | 61–108 | 89.81 |  | 0.9942 |  |
| 4 | 60–119 | 97.47 |  | 0.9930 |  |
| 5 | 113–197 | 160.06 |  | 0.9923 | Hygrothermal ageing  (30% RH) |
| 6 | 98–162 | 142.27 |  | 0.9911 |  |
| 7 | 100–118 | 111.13 |  | 0.9969 |  |
| 8 | 94–128 | 118.36 |  | 0.9937 |  |
| 9 | 132–244 | 152.78 |  | 0.9879 | Hygrothermal ageing  (70% RH) |
| 10 | 123–188 | 133.47 |  | 0.9958 |  |
| 11 | 121–207 | 131.59 |  | 0.9955 |  |
| 12 | 27–323 | 173.39 |  | 0.9608 |  |
| 13 | 135–174 | 150.23 |  | 0.9885 | Hygrothermal ageing  (100% RH) |
| 14 | 161–238 | 192.73 |  | 0.9796 |  |
| 15 | 159–251 | 208.22 |  | 0.9899 |  |
| 16 | 167–302 | 221.26 |  | 0.9975 |  |
| Ozawa method | | | | | |
| 0 | 145–176 | 152.79 | 212.52^36^ | 0.9970 | Unaged |
| 1 | 109–147 | 140.40 |  | 0.9991 | Thermal ageing |
| 2 | 133–165 | 142.07 |  | 0.9967 |  |
| 3 | 100–126 | 103.48 |  | 0.9980 |  |
| 4 | 109–137 | 112.55 |  | 0.9991 |  |
| 5 | 132–194 | 161.65 |  | 0.9968 | Hygrothermal ageing  (30% RH) |
| 6 | 124–168 | 139.15 |  | 0.9962 |  |
| 7 | 119–143 | 125.51 |  | 0.9995 |  |
| 8 | 127–158 | 136.37 |  | 0.9960 |  |
| 9 | 151–221 | 160.60 |  | 0.9957 | Hygrothermal ageing  (70% RH) |
| 10 | 139–190 | 151.97 |  | 0.9976 |  |
| 11 | 138–213 | 155.10 |  | 0.9955 |  |
| 12 | 95–212 | 151.53 |  | 0.9927 |  |
| 13 | 143–170 | 160.05 |  | 0.9953 | Hygrothermal ageing  (100% RH) |
| 14 | 187–220 | 203.08 |  | 0.9788 |  |
| 15 | 218–257 | 245.35 |  | 0.9922 |  |
| 16 | 189–242 | 205.46 |  | 0.9988 |  |

**Table S2.** Activation energies for all samples, calculated using the Friedman and Ozawa methods.

| Ageing type | Root square (*r*^2^) | Constant coefficients | Standard error | Empirical models |
| --- | --- | --- | --- | --- |
| Thermal | 0.9211 | $\varphi_{0}$= 2.6248, $\varphi_{1}$ = -0.0158 | 0.0739,  0.0023 | Logarithmic |
| Hygrothermal  (30% RH) | 0.9998 | $\varphi_{0}$ = 0.7918, $\varphi_{1}$ = 0.2080, $\varphi_{2}$ = -0.4593 | 0.0010, 0.0016, 0.0086 | Exponential type 1 |
| Hygrothermal (70% RH) | 0.9692 | $\varphi_{0}$ = 0.6338, $\varphi_{1}$ = 0.3553, $\varphi_{2}$ = -0.1179 | 0.0578, 0.0535, 0.0415 | Exponential type 1 |
| Hygrothermal (100% RH) | 0.8417 | $\varphi_{0}$ = 0.0062, $\varphi_{1}$ = -0.0674, $\varphi_{2}$ = 0.0027 | 0.0515, 0.0248, 0.0020 | Exponential type 2 |

**Table S3.** Parameters and standard errors for all ageing cases, used to predict the heat degradation ratio of aged ZPP.
